# Treatment outcomes in patients with opioid use disorder initiated by prescription: a systematic review protocol

**DOI:** 10.1186/s13643-018-0682-0

**Published:** 2018-01-25

**Authors:** Nitika Sanger, Meha Bhatt, Laura Zielinski, Stephanie Sanger, Hamnah Shahid, Bianca Bantoto, M.Constantine Samaan, Russell de Souza, Zainab Samaan

**Affiliations:** 1 Medical Sciences Graduate Program, McMaster University, Hamilton, ON Canada; 2 Department of Health Research Methods, Evidence and Impact, McMaster University, Hamilton, ON Canada; 3 Department of Psychiatry and Behavioural Neurosciences, McMaster University, Hamilton, ON Canada; 4 Health Science Library, McMaster University, Hamilton, ON Canada; 5 Arts & Sciences, McMaster University, Hamilton, ON Canada; 6 Integrated Sciences Program, McMaster University, Hamilton, ON Canada; 7 Department of Pediatrics, McMaster University, Hamilton, ON Canada; 8 Division of Pediatric Endocrinology, McMaster Children’s Hospital, Hamilton, ON Canada; 9 Peter Boris Centre for Addictions Research, St. Joseph’s Healthcare Hamilton, Hamilton, ON Canada; 10 Mood Disorders Program, St. Joseph’s Healthcare Hamilton, 100 West 5th St., Hamilton, ON L8N 3K7 Canada

**Keywords:** Opioid substitution therapy, OST, Prescription opioids, Systematic review, Opioid use disorder, Protocol

## Abstract

**Background:**

In North America, opioid use has become a public health crisis with policy makers declaring it a state of emergency. Opioid substitution therapy (OST) is a harm-reduction method used in treating opioid use disorder. While OST has shown to be successful in improving treatment outcomes, there is still a great degree of variability among patients. This cohort of patients has shifted from young males using heroin to a greater number of older people and women using prescription opioids. The primary objective of this review is to examine the literature on the association between the first exposure to opioids through prescription versus illicit use and OST treatment outcomes.

**Method:**

An electronic search will be conducted on the EMBASE, MEDLINE, PsycINFO, and Cumulative Index to Nursing and Allied Health Literature (CINAHL) databases. Two independent reviewers will conduct the initial title and abstract screenings using predetermined criteria for inclusion and exclusion. Reviewers will then conduct full-text data extraction using a pilot-tested data extraction form in duplicate. A third author will resolve disagreements if consensus cannot be reached. Quality and risk of bias assessment will be conducted along with a sensitivity analysis for all included studies. Qualitative summary of the evidence will be provided, and when possible, a meta-analysis will be conducted, along with heterogeneity calculation. The reporting of this protocol follows the PRISMA-P.

**Discussion:**

We expect that this review will help determine whether patients that were initially exposed to opioids through a prescription differ in OST treatment outcomes in comparison to people who used opioids through illicit means. We hope that this review will provide evidence related to prescription opioids exposure and future treatment outcomes, which will aid clinicians in their decisions to prescribe opioids or not for specific populations at risk.

**Systematic review registration:**

PROSPERO CRD42017058143

**Electronic supplementary material:**

The online version of this article (10.1186/s13643-018-0682-0) contains supplementary material, which is available to authorized users.

## Background

### Rationale

The global opioid crisis is marked by a striking 32 to 36 million individuals who used opioids worldwide [1]. Illicit opioid use is associated with an increased risk of infections such as HIV and hepatitis C, dependency, poly-substance use, psychiatric comorbidity, criminal activity and death [2–4]. Opioids are now the primary cause of drug-related deaths in North America, with a 200% increase in the number of opioid-related deaths since 2000 [5]. Regular use of opioids can result in opioid use disorder (OUD), a chronic psychiatric disorder characterized by loss of control over the drug use, behavioural and psychological symptoms related to drug use and impairment in normal function of the affected individuals [2]. Treatment of OUD also takes an economic toll on the healthcare system [6]. The increased misuse of prescription opioids has contributed to these rising numbers of opioid use and its related consequences [5]. Historically, many individuals were first introduced to opioids through recreational drugs such as heroin [7, 8]. However, recent opioid use patterns have contributed to a demographic shift in which individuals developed OUD after being exposed to opioids by means of prescription drugs such as fentanyl, codeine, or oxycodone [9, 10]. Today, Canada is the world’s second highest consumer of prescription opioids after USA [11].

Currently, opioid substitution therapy (OST) is used to treat OUD. OST is a harm reduction treatment that aims to limit adverse risks and events associated with illicit opioid use [12]. This entails the prescription of longer-acting opioids with less euphoric effects in order to minimize cravings and prevent withdrawal symptoms [12, 13]. The most commonly used opioid substitutes are methadone, buprenorphine, naltrexone and suboxone® (a combination of buprenorphine and naloxone) [12–14]. OST has a positive impact on OUD including a variety of social and health-related factors, such as a decline in the use of illicit substances, unemployment, HIV prevalence, criminal activities and mortality [2, 13, 15]. OST has also demonstrated improved social functioning and treatment retention [13–15]. However, while OST has demonstrated some success in managing OUD, there is still a great degree of variability in treatment outcomes [4].

This variability in treatment outcomes may be partially explained by a shifting OST population resulting from changes in the way in which an individual is first introduced to opioids. A recent study estimated that 52% of women and 38% of men are seeking treatment for OUD having first been exposed to opioids through a prescription [9]. Previous research demonstrates that patients in treatment for OUD were mainly young adult males, around 20 years of age, who injected heroin [8, 9, 16]. However, the patients receiving OST today are older and have a greater number of women [10, 17, 18]. This demographic shift warrants new investigation, as past research many no longer apply to this population.

Studies that look at the relationship between patients who initially started misusing opioids through a medical prescription and OST outcomes present conflicting findings. Some studies show that those in buprenorphine treatment that have misused prescriptions only have better treatment retention in comparison to people who have misused heroin [19] while other studies demonstrate that those that have misused prescriptions only do not differ in treatment retention from those misusing illicit opioids such as heroin [20].

The relationship between prescription opioids and OST outcomes may also be affected by physical health status. Opioids have become one of the most commonly used medications for pain in North America due to their analgesic effects [21]. Given the high prevalence of comorbid pain in the OUD population, it has been suggested that the chronic pain population is at risk for an increased likelihood to misuse prescription opioids [21–23].

It remains unclear, however, as to whether an association between initial exposure to opioids through a medical prescription and OST outcomes exists and if confounding variables heavily influence this relationship. Conducting a systematic evaluation of the literature on this topic is essential and can identify factors influencing treatment outcomes that may be overlooked in individual studies. We hypothesize that patients that were exposed to opioids through a prescription will have a different response to OST as defined by illicit opioid use and treatment retention.

## Objectives

The aim of this systematic review is to synthesize and appraise the existing literature on the effects of initial exposure to opioids by prescription compared to those introduced through illicit opioid substitution therapy treatment outcomes in patients diagnosed with opioid use disorder.

Specifically, the study objectives are:

1. Summarize the literature examining the association between exposure to opioids through a medical prescription and OST outcome (primary: illicit opioid use, secondary: treatment retention and poly-substance use).
2. If possible, combine study findings in a meta-analysis comparing the OST treatment outcomes of those that were initially exposed to opioids through a legitimate prescription and those that were introduced through illicit means.
3. Conduct subgroup analyses based on age, sex, country and method of OST treatment outcome measurement.

## Methods

### Eligibility criteria

This review will consist of published observational cross-sectional and cohort studies and randomized control trials (RCTs) examining the association between opioid prescription misuse and OST outcomes. These studies may have been conducted in different settings including hospital, outpatient or community-based. Primary studies will include the main exposure to opioids through a prescription and OST treatment outcomes. The included studies will be comparing those introduced to opioids through a legitimate prescription and those introduced through illicit means. The individuals that began their use through a prescription not prescribed to them will be in the group of those that obtained opioids through other means (i.e. a family member, street or friend) as this can be defined as illicit use. There will be no age, sex, language or type of study population restrictions.

Studies will be excluded if they do not assess at least one of the primary or secondary outcomes of interest detailed below. Most of the research on OST treatment outcomes study the current type of opioid misuse (i.e. street drugs or prescription) and fail to identify the method of initial exposure to opioids. As such, these studies will be omitted from our analysis, as it will not be possible to make conclusions pertaining to the primary exposure of interest and the association with OST results. In addition, studies investigating patients in OST for other reasons apart from treatment of OUD will also be omitted.

### Outcomes and prioritization

The primary study outcome, illicit opioid use, will be used to determine the effectiveness of the OST and may be quantified in various ways such as urine toxicology or self-reports as provided in the primary studies. Secondary outcomes will include treatment retention and poly-substance use. Treatment retention may be quantified as ratio of people who are still in treatment at the time the study completion or average period of time in treatment. Poly-substance use may be measured in similar ways to illicit opioid use (i.e. urine toxicology, self-reports).

### Information sources

In order to identify the relevant articles that will be used in the review, a health sciences librarian (SS) was consulted to develop a search strategy. The databases to be searched from inception are EMBASE, MEDLINE, PsycINFO, and Cumulative Index to Nursing and Allied Health Literature (CINAHL). Articles will be identified using search terms related to prescription opioids and opioid use disorder together with their medical subject headings (MeSH) in different combinations (Table 1). An in-depth search will be carried out comprising of keywords found in the title, and abstract fields. To ensure that unnecessary restrictions on the search findings are avoided, the study findings will not be included in the search strategy. The searches will be restricted to studies conducted in human research participants. Gray literature will be searched using ProQuest Dissertations as well as the Theses Worldwide database. Lastly, a comprehensive hand search of reference lists of the relevant articles will be carried out to identify additional articles that may not have been captured in the original search.

**Table 1:** Search strategy

MEDLINE = 6250	1 exp Analgesics, Opioid/ 2 (opiate* or opioid* or fentanyl or narcotic* or dilaudid or oxycontin* or oxycod*).ti,ab. 3 1 or 2 4 exp Drug Prescriptions/ 5 (prescript* or prescrib* or pharmaceutical* or legal*).ti,ab. 64 or 5 73 and 6 8 ((prescript* or prescrib* or pharmaceutical*) adj2 (opioid* or opiate* or dilaudid or fentanyl or codeine or oxyco*)).ti,ab. 97 or 8 10 Opioid-Related Disorders/ 11 Heroin Dependence/ 12 Substance-Related Disorders/ 13 Substance Abuse, Intravenous/ 14 ((opiate* or opioid* or heroin* or oxyco* or codeine* or dilaudid or fentanyl or drug* or substance*) adj2 (use* or using or misuse* or abus* or dependence* or dependent* or addict*)).ti,ab. 1510 or 11 or 12 or 13 or 14 169 and 15 17 exp animals/ not (humans/ and exp animals/) 1816 not 17
EMBASE = 14,649	1 exp heroin dependence/ 2 opiate/ 3 exp opiate addiction/ 4 substance abuse/ 5 ((opiate* or opioid* or heroin* or oxyco* or codeine* or dilaudid or fentanyl or drug* or substance*) adj2 (use* or using or misuse* or abus* or dependence* or dependent* or addict*)).ti,ab. 61 or 2 or 3 or 4 or 5 7 ((prescript* or prescrib* or pharmaceutical*) adj2 (opioid* or opiate* or dilaudid or fentanyl or codeine or oxyco*)).ti,ab. 8 (prescript* or prescrib* or pharmaceutical* or legal*).ti,ab. 9 exp prescription/ 10 exp prescription drug/ 11 (opiate* or opioid* or fentanyl or narcotic* or dilaudid or oxycontin* or oxycod*).ti,ab. 12 exp narcotic analgesic agent/ 13 11 or 12 148 or 9 or 10 1513 and 14 167 or 15 176 and 16 18 limit 17 to human
PsycINFO = 2898	1 exp Opiates/ 2 (opiate* or opioid* or fentanyl or narcotic* or dilaudid or oxycontin* or oxycod*).ti,ab. 3 exp Prescription Drugs/ 41 or 2 5 (prescript* or prescrib* or pharmaceutical* or legal*).ti,ab. 63 or 5 74 and 6 8 ((prescript* or prescrib* or pharmaceutical*) adj2 (opioid* or opiate* or dilaudid or fentanyl or codeine or oxyco*)).ti,ab. 97 or 8 10 exp Heroin Addiction/ or exp Heroin/ 11 exp Intravenous Drug Usage/ 12 ((opiate* or oxyco* or opioid* or heroin* or codeine* or dilaudid or fentanyl or drug* or substance*) adj2 (use* or using or misuse* or abus* or dependence* or dependent* or addict*)).ti,ab. 1310 or 11 or 12 149 and 13
CINAHL = 1143	1 (MH “Drugs, Non-Prescription”) OR (MH “Drugs, Prescription”) OR (MH “Prescriptions, Drug”) OR (MH “Drugs, Off-Label”) 2 (MH “Substance Use Disorders”) 3 (MH “Heroin”) OR (MH “Substance Dependence”) 4 (MH “Substance Abuse, Intravenous”) 5 ((opiate* or opioid* or oxyco* or heroin* or codeine* or dilaudid or fentanyl or drug* or substance*) N2 (use* or using or misuse* or abus* or dependence* or dependent* or addict*)) 6 (MH “Analgesics, Opioid”) 7 (opiate* or opioid* or fentanyl or narcotic* or dilaudid or oxycontin* or oxycod*) 82 OR 3 OR 4 OR 5 9 ((prescript* or prescrib* or pharmaceutical*) n2 (opioid* or opiate* or dilaudid or fentanyl or codeine or oxyco*)) 101 OR 6 OR 7 11 (prescript* or prescrib* or pharmaceutical* or legal*) 121 OR 11 1310 AND 12 149 OR 13 158 AND 14 (limtiters- human)

### Search strategy

#### Study records

##### Data management

Articles identified by the search strategy will be uploaded to an online platform known as Google Forms. Google Forms will allow for management of the articles and will also allow the authors to collaborate simultaneously. The review team will be provided training on how to use Google Forms prior to the commencement of the study to ensure calibration of the forms and the data abstraction methods. A pilot of 20 studies will first be carried out to calibrate the study forms and assess level of agreement.

##### Selection process

Two independent reviewers will carry out the title and abstract screening in duplicate to identify appropriate articles using previously established criteria. Eligible articles will then undergo full-text review in duplicate. Disagreements will be resolved by discussion and consensus, and in cases were no resolution is reached, a third author will be consulted. During each stage of screening, a kappa statistic will be used to establish inter-rater agreements. Exceptional agreement between reviewers will be demonstrated as a kappa value of at least 0.75 [24]. In cases were additional clarification is needed, the primary study authors will be contacted to help determine eligibility. The preferred reporting items for systematic reviews and meta-analyses (PRISMA) [25] flow diagram will be used when reporting the full systematic review.

##### Data collection process

Independent reviewers will retrieve the data using a previously piloted data extraction form in duplicate (see Additional file 1). To ensure standardization, consistency among reviewers will be addressed by assessing completed pilot data extraction forms.

##### Data items

For included data items, please see Additional file 1. The information to be retrieved by the reviewers will consist of details of the publication such as name of the first author, year of publication, journal and country of publication; research design that was used; demographics of the research participants; type and method of measuring opioid exposure (i.e. medical prescription or illicit); OST outcome measures; overall findings of the study and the study statistical results. In the case of missing data for any study, the authors will be contacted.

##### Risk of bias

The risk of bias will be appraised using the modified Newcastle-Ottawa Scale (NOS) [26, 27] to appraise the likelihood of bias in studies that are mainly observational in nature. This modified scale comprises seven questions that assess bias in four realms: choice bias, performance bias, identification bias and information bias. Risk of bias is quantified on a scale 0 to 3 where 0 is high risk and 3 is low risk. The modified model has eliminated items concerning the comparability of groups. To assess risk of bias in RCTs, we will use the Cochrane Collaboration tool which will look at six domains including selection bias, reporting bias, attrition bias, performance bias, detection bias and other biases [28]. These results will be displayed in a table to facilitate easy comparison between the quality of studies included in this review.

##### Data synthesis

All included studies will be appraised with a qualitative summary, and then if possible, a meta-analysis will be undertaken. Our primary analysis will compare treatment outcomes for patients that initiated opioid use by prescription (and continue to use prescription opioids) to those patients that started using opioids through illicit means. If studies further report that the patients who initially began through prescription have transitioned to using non-prescription opioids (or both), we will conduct a sensitivity analysis by removing these studies to determine whether it has an effect on the outcomes. Studies will be merged in a meta-analysis depending on the similarity between design of the study and the measurements of the outcomes. Depending on the design of the research, direct estimates will be pooled separately as pooling data from observational studies as well as RCTs is not advisable [29].

To account for the anticipated heterogeneity in the included studies, a random effect model for the meta-analysis will be used. This model takes both within-study and between-study variance into consideration to offer a modest estimate in comparison to a fixed-effect model. The outcomes will be featured on a forest plot. Moreover, a sensitivity analysis might also be carried out to compare the outcomes of the studies with high or low risk of bias.

Heterogeneity will be computed among the pooled articles through the use of *I*^2^ statistic. It is recommended that cut-off values are not enforced since the significance of heterogeneity relies on a variety of factors, although Cochrane has recommended that a value of < 40% might not signify a noteworthy amount of heterogeneity [29]. Therefore, likely sources of heterogeneity are going to be evaluated as long as there is an *I*^2^ statistic > 40%. In this case, subgroup analyses will also be conducted.

Some of the likely sources of heterogeneity include age, sex, types of opioids and outcome measurements. These are going to be examined through the use of subgroup analyses. We also plan to conduct a subgroup analysis if possible examining the differences in treatment outcomes for individuals who obtained opioids through different sources (i.e. street, family members, friend).

##### Meta-bias

Egger’s plot will be created to assess the likelihood of publication bias in the included articles.

##### Confidence in the cumulative evidence

The grading of recommendations, assessment, development and evaluation (GRADE) framework will be used to assess the quality of the evidence [30]. This scale evaluates evidence based on five realms: risk of bias, publication bias, consistency, directness and accuracy.

##### Presenting and reporting of the study results

This systematic review will be reported in compliance with PRISMA reporting guidelines [25]. A flow diagram will be used to demonstrate the selection of studies including reasons for exclusion. The present protocol follows the preferred reporting items for systematic review and meta-analysis protocol (PRISMA-P) guidelines which is attached (see Additional file 2) [31].

## Discussion

Using the evidence obtained from this systematic review, we expect to draw conclusions regarding the presence of an association between being exposed to opioids through a medical prescription and opioid substitution therapy outcomes. Examining the current literature in a systematic way will allow us to summarize existing findings on this topic and to critically appraise the risk of bias and methodological quality of these studies. The present literature primarily focused on the cohort of patients that were exposed to opioids through illicit means and little is known about the cohort of patients that started misusing opioids after using a prescription. This new shift in demographic profile of opioid users and the predominance of prescription opioid use over heroin in different parts of the world including Canada and the USA, the highest opioid-consuming countries in the world, warrants a detailed examination of the literature.

Given the rise of prescription opioid use in Canada and the USA, it is important that we evaluate factors that may affect the effectiveness of opioid substitution treatment for this cohort of patients.

## Additional files

Additional File 1: Data extraction form in. This form includes all the information we intend to extract from the included studies. (PDF 54 kb)

Additional File 2: PRISMA-P checklist. These are the guidelines that this protocol was reported by. (PDF 92 kb)

## Abbreviations

GRADE: Grading of Recommendations Assessment, Development and Evaluation
NOS: Newcastle-Ottawa scale
OST: Opioid substitution therapy
OUD: Opioid use disorder
PRISMA-P: Preferred reporting items for systematic review and meta-analysis protocols
PRISMA: Preferred reporting items for systematic reviews and meta-analyses
RCT: Randomized control trials

## References

[1] UNODC. World drug report 2015 [Internet], vol. 53: United Nations publication; 2015. p. 1689–99. Available from: https://www.unodc.org/documents/wdr2015/World_Drug_Report_2015.pdf.

[2] American Psychiatric Association, 2013. Diagnostic and statistical manual of mental disorders (DSM-5®). American Psychiatric Pub. Washington, DC.

[3] Fischer B, Rehm J, Brissette S, Brochu S, Bruneau J, El-Guebaly N (2005). Illicit opioid use in Canada: comparing social, health, and drug use characteristics of untreated users in five cities (OPICAN study). J Urban Heal.

[4] Bertschy G (1995). Methadone maintenance treatment: an update. Eur Arch Psychiatry Clin Neurosci.

[5] Rudd RA, Aleshire N, Zibbell JE, Gladden RM (2016). Increases in drug and opioid overdose deaths—United States, 2000–2014. Centers Dis Control Prev MMWR.

[6] Degenhardt L, Hall W (2012). Extent of illicit drug use and dependence, and their contribution to the global burden of disease. Lancet.

[7] Unick GJ, Rosenblum D, Mars S, Ciccarone D (2013). Intertwined epidemics: national demographic trends in hospitalizations for heroin- and opioid-related overdoses, 1993–2009. PLoS One [Internet].

[8] Mattick RP, Breen C, Kimber J, Davoli M. Methadone maintenance therapy versus no opioid replacement therapy for opioid dependence. Cochrane Database Syst Rev. 2009;3:CD002209. 10.1002/14651858.CD002209.pub2 PMC7097731 19588333

[9] Bawor M, Dennis BB, Varenbut M, Daiter J, Marsh DC, Plater C (2015). Sex differences in substance use, health, and social functioning among opioid users receiving methadone treatment: a multicenter cohort study. Biol Sex Differ.

[10] Zhong W, Maradit-Kremers H, Sauver JLS, Yawn BP, Ebbert JO, Roger VL, Jacobson DJ, McGree ME, Brue SM, Rocca WA. Age and sex patterns of drug prescribing in a defined American population. In Mayo Clinic Proceedings (Vol. 88, No. 7, pp. 697-707). Elsevier; 2013. 10.1016/j.mayocp.2013.04.021 PMC3754826 23790544

[11] Darnall BD, Stacey BR, Chou R (2012). Medical and psychological risks and consequences of long-term opioid therapy in women. Pain Med.

[12] Schuckit MA (2016). Treatment of opioid-use disorders. N Engl J Med.

[13] Darke S, Marel C, Slade T, Ross J, Mills KL, Teesson M (2015). Patterns and correlates of sustained heroin abstinence: findings from the 11-year follow-up of the Australian treatment outcome study. J Stud Alcohol Drugs.

[14] Wright NMJ, Sheard L, Adams CE, Rushforth BJ, Harrison W, Bound N (2011). Comparison of methadone and buprenorphine for opiate detoxification (LEEDS trial): a randomised controlled trial. Br J Gen Pr.

[15] Park TW, Cheng DM, Lloyd-Travaglini CA, Bernstein J, Palfai TP, Saitz R (2015). Changes in health outcomes as a function of abstinence and reduction in illicit psychoactive drug use: a prospective study in primary care. Addiction.

[16] Li Y, Kantelip J-P, Gerritsen-van Schieveen P, Davani S (2008). Interindividual variability of methadone response. Mol Diagn Ther.

[17] Hurley RW, MCB A (2008). Sex, gender, and pain: an overview of a complex field. Anesth Analg.

[18] Simoni-Wastila L, Ritter G, Strickler G (2004). Gender and other factors associated with the nonmedical use of abusable prescription drugs. Subst Use Misuse.

[19] Moore BA, Fiellin DA, Barry DT, Sullivan LE, Chawarski MC, O’Connor PG (2007). Primary care office-based buprenorphine treatment: comparison of heroin and prescription opioid dependent patients. J Gen Intern Med.

[20] Nielsen S, Hillhouse M, Mooney L, Fahey J, Ling W (2012). Comparing buprenorphine induction experience with heroin and prescription opioid users. J Subst Abus Treat.

[21] Setnik B, Roland CL, Sommerville KW, Pixton GC, Berke R, Calkins A (2015). A multicenter, primary care-based, open-label study to identify behaviors related to prescription opioid misuse, abuse, and diversion in opioid-experienced patients with chronic moderate-to-severe pain. J Pain Res.

[22] Højsted J, Ekholm O, Kurita GP, Juel K, Sjøgren P (2013). Addictive behaviors related to opioid use for chronic pain: a population-based study. Pain.

[23] Dennis BB, Samaan MC, Bawor M, Paul J, Plater C, Pare G (2014). Evaluation of clinical and inflammatory profile in opioid addiction patients with comorbid pain: results from a multicenter investigation. Neuropsychiatr Dis Treat.

[24] Orwin RG, Vevea JL (2009). Evaluating coding decisions. Handb Res Synth meta-analysis.

[25] Moher D, Liberati A, Tetzlaff J, Altman DG, Group P (2009). Preferred reporting items for systematic reviews and meta-analyses: the PRISMA statement. PLoS Med.

[26] Wells GA, Shea B, O’connell D, Peterson J, Welch V, Losos M, et al. The Newcastle-Ottawa Scale (NOS) for assessing the quality if nonrandomized studies in meta-analyses. 2009. Epub Available from URL http//www ohri ca/programs/clinical_epidemiology/oxford htm [cited 2009 Oct 19]. 2013;

[27] Bawor M, Dennis BB, Anglin R, Steiner M, Thabane L, Samaan Z (2014). Sex differences in outcomes of methadone maintenance treatment for opioid addiction: a systematic review protocol. Syst Rev..

[28] Higgins JPT, Altman DG, Gøtzsche PC, Jüni P, Moher D, Oxman AD (2011). The Cochrane Collaboration’s tool for assessing risk of bias in randomised trials. BMJ.

[29] Higgins JPT, Green S. Cochrane Handbook for Systematic Reviews of Interventions Version 5.1.0 [updated March 2011]. The Cochrane Collaboration. 2011. Available from http://handbook.cochrane.org.

[30] Guyatt GH, Oxman AD, Schünemann HJ, Tugwell P, Knottnerus A (2011). GRADE guidelines: a new series of articles in the Journal of Clinical Epidemiology. J Clin Epidemiol.

[31] Moher D, Shamseer L, Clarke M, Ghersi D, Liberati A, Petticrew M (2015). Preferred reporting items for systematic review and meta-analysis protocols (PRISMA-P) 2015 statement. Syst Rev.

