# Supplementary material for: Treatment outcomes in patients with opioid use disorder initiated by prescription: a systematic review protocol
Source: Syst Rev. 2018 Jan 25;7:16. doi: 10.1186/s13643-018-0682-0 (PMC5784712; doi:10.1186/s13643-018-0682-0)
Supplement: Supplementary file 1 — Data extraction form in. This form includes all the information we intend to extract from the included studies. (PDF 54 kb) [file 13643_2018_682_MOESM1_ESM.pdf]

Data Extraction Form

Study ID: _____________ Reviewer Initials: ________

Publication Details

Author (last name, first initial): ______________________________ Year: ________________
Title: ____
Journal: ___________________________________ Country: ___________________________

Methods

Study design: _____________________ Study setting: _________________________________
Length of study: ______________________
Description of sample:

____
Exposure: ________________________________ Intervention (if applicable): ______________

Demographics

Number of participants: Total: _____ Men: _____ Women: ____Other: ____ Per group: ______
Mean age (SD): Total: ___________ Men: _________ Women: __________ Other: _______
Per group: ____
Ethnicity:

____

Exposure measurements (Initial exposure to opioids). Circle one.

Self-report Other ______________________________
Comments:

____

Outcome measurements:

Illicit opioid use: ____
Treatment retention: ____
Poly-substance use:____
Comments:

____

Results

Statistical methods: ______________________________ Adjusted for: ___________________
Coefficient: ______________________95% CI: _______________________ p-value: _______
Findings: ____
Limitations: ____

Inclusion Criteria

Patients on opioid substitution therapy

Initial exposure to opioids (i.e. medical prescription or illicit) measured

RCT or observational study design

Exclusion Criteria

OST being used for purposes other than maintenance treatment (i.e. recreational or detoxification)

Study does not measure a primary (illicit opioid use) or secondary (treatment retention and poly-substance use) outcome variables

Additional Comments:
